# lncRNA RMST is associated with the progression and prognosis of gastric cancer via miR-204-5p

**DOI:** 10.1186/s13008-024-00117-x

**Published:** 2024-04-12

**Authors:** Huimei Cai, Chenhui Li, Zhou Wu

**Affiliations:** 1 Department of Gastroenterology, Affiliated Fuzhou First Hospital of Fujian Medical University, No. 190, Dadao Road, Taijiang District, Fuzhou, 350000 China; 2 Department of Clinical Medicine, Fujian Medical University, Fuzhou, 350122 China

**Keywords:** Gastric cancer, lncRNA RMST, miR-204-5p, Tumor progression, Disease development, Prognosis

## Abstract

**Background:**

Exploring novel biomarkers for gastric cancer holds promise for enhancing patients’ therapy and survival rates. lncRNAs and miRNAs have emerged as important biomarkers for various human cancers. However, the role of lncRNA RMST (RMST) in gastric cancer development and the mechanism underlying its function remains unclear.

**Results:**

Significant upregulation of RMST was observed in gastric cancer tumor tissues. RMST levels showed strong correlation with patients’ lymph node metastasis and TNM stage and serving as a predictor of adverse prognosis RMST negatively regulated miR-204-5p, which in turn mediated the inhibitory effects of RMST knockdown on gastric cancer cell growth and metastasis.

**Conclusion:**

RMST served as both a prognostic biomarker and tumor promoter by modulating miR-204-5p. Inhibiting RMST could represent a novel and potential therapeutic strategy for gastric cancer.

**Supplementary Information:**

The online version contains supplementary material available at 10.1186/s13008-024-00117-x.

## Background

Gastric cancer ranks among the most prevalent malignant tumors worldwide, contributing significantly to cancer-related mortality [1, 2]. Surgery remains the primary treatment for patients with gastric cancer, however, many patients in advanced stages miss the optimal time window for surgical intervention [3]. Despite considerable advancement in therapeutic approaches for gastric cancer, the clinical outcomes of patients remain poor [4, 5]. The absence of obvious symptoms during the development of gastric cancer largely contributes to its poor prognosis. Therefore, exploring effective biomarkers to monitor tumor progression and disease development is pivotal in improving treatment efficacy for gastric cancer.

Studies have shown that long non-coding RNAs (lncRNAs) regulate gene expression at the post-transcriptional level, the expression of target coding genes, and the silencing of the transcriptional gene [6, 7]. The onset and progression of gastric cancer are complicated processes involving cell differentiation, growth, metastasis, and apoptosis, with lncRNAs playing pivotal roles [8]. Dysregulation of lncRNAs emerged as an indicator of clinical significance. LncRNA rhabdomyosarcoma 2-associated transcript (lncRNA RMST, RMST) has been identified among the top ten survival-associated lncRNAs in gastric cancer due to its elevated expression [9], with reported biological effects on other malignant tumors. For example, RMST has been implicated as a suppressor in triple-negative breast cancer, where it inhibits cell viability and promotes cell apoptosis [10]. However, limited data exists demonstrating the specific role of RMST in gastric cancer. This study aims to address the knowledge gap.

In mechanism, lncRNAs typically display their functional roles by regulating downstream microRNAs (miRNAs). Several miRNAs have been associated with RMST. For instance, miR-204-5p was identified as a direct target of RMST in mediating oxygen-glucose deprivation-induced brain injury [11]. Moreover, miR-204-5p was shown to inhibit tumor progression and trigger apoptosis in gastric cancer cells [12]. Modulating miR-204-5p was hypothesized as the mechanism underlying the potential functional role of RMST in gastric cancer.

## Results

### Dysregulation of RMST in gastric cancer

**Fig. 1:**
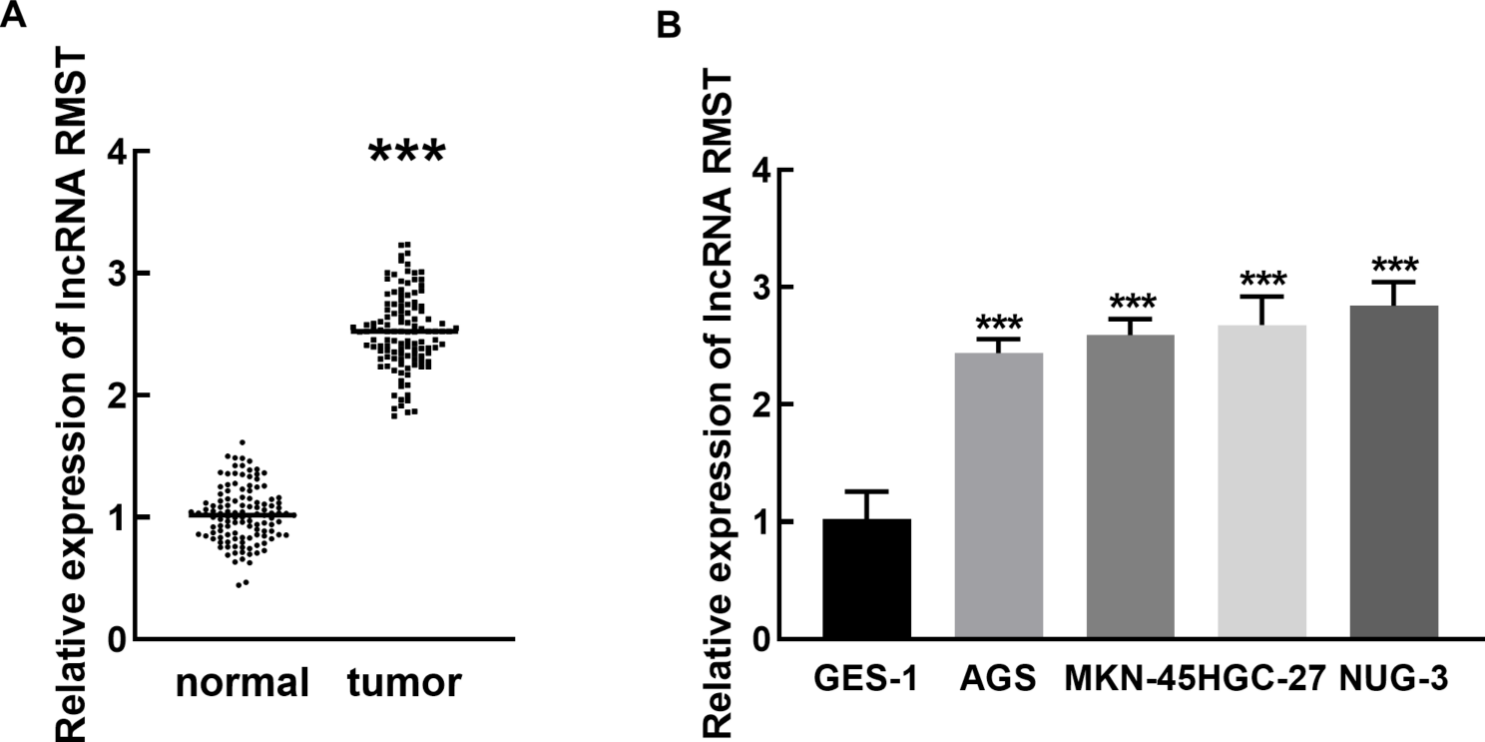
Expression of RMST in gastric cancer tissues (**A**) and cells (**B**). ***P < 0.001

RMST exhibited significant upregulation in tumor tissues compared with normal tissues (*P* < 0.001, Fig. 1A). Consistently, elevated RMST was also observed in gastric cancer cells (AGS, MKN-45, HGC-27, and NUG-3 cells) relative to the normal gastric mucosa cell, GES-1 cell(*P* < 0.001), and no significant differences were observed among the four gastric cancer cells *P* > 0.05, Fig. 1B). AGS and MKN45 cells are commonly used in gastric cancer research, therefore, these two cells were randomly selected for the subsequent in vitro experiments.

### Clinical significance of RMST in gastric cancer

Based on the average expression of RMST in tumor tissues, the patients included in the study were divided into a high-RMST group consisting of 40 males and 24 females, and a low-RMST group comprising 34 males and 23 females. The expression of RMST was significantly associated with the lymph node metastasis status (*P* = 0.017) and TNM stage (*P* = 0.021) of patients (Table 1). Specifically, patients with positive lymph node metastasis status and advanced TNM stage showed relatively higher expression of RMST.

**Table 1:** Association of RMST with the major clinical features of gastric cancer patients

	Total (*n* = 121)	RMST expression		P value
		Low (*n* = 57)	High (*n* = 64)	
Age (years)				0.444
< 60	66	29	37	
≥ 60	55	28	27	
Gender				0.748
Male	74	34	40	
Female	47	23	24	
Tumor size (cm)				0.217
< 4	65	34	31	
≥ 4	56	23	33	
LNM				0.017
Negative	78	43	35	
Positive	43	14	29	
TNM stage				0.021
I-II	83	45	38	
III	38	12	26	
Histological type				0.372
Intestinal	67	34	33	
Diffuse	54	23	31	

LNM: lymph node metastasis

**Fig. 2:**
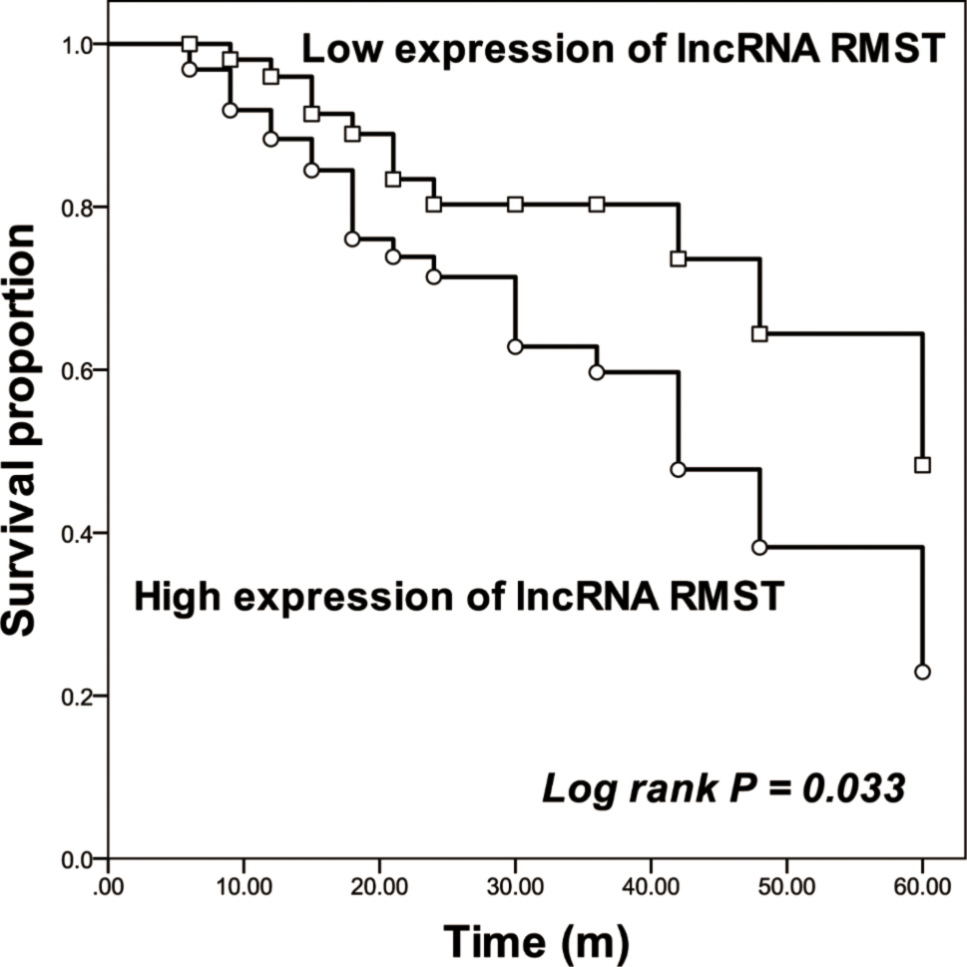
Kaplan-Meier curves of enrolled patients based on the expression of RMST. log rank *P* = 0.033

On the other hand, the median survival of enrolled gastric cancer patients were 48 months. Patients in the high-RMST groups possessed a superior overall survival rate than patients in the low-RMST group (log-rank *P* = 0.033, Fig. 2). Furthermore, RMST (HR = 2.259, 95% CI = 1.046–4.882, *P* = 0.038, Table 2) was identified as an independent prognostic factor for gastric cancer.

**Table 2:** Prognostic value of RMST and clinical features evaluation by Cox regression analysis

	95% CI	HR value	P value
RMST	1.046–4.882	2.259	0.038
Age	0.736–3.492	1.603	0.235
gender	0.754–3.171	1.546	0.234
Tumor size	0.746-3.100	1.521	0.249
LNM	0.640–2.902	1.363	0.421
TNM stage	1.020–4.621	2.171	0.044
Histological type	0.852–4.389	1.934	0.115

LNM: lymph node metastasis

### The biological effect of RMST and its potential mechanism

**Fig. 3:**
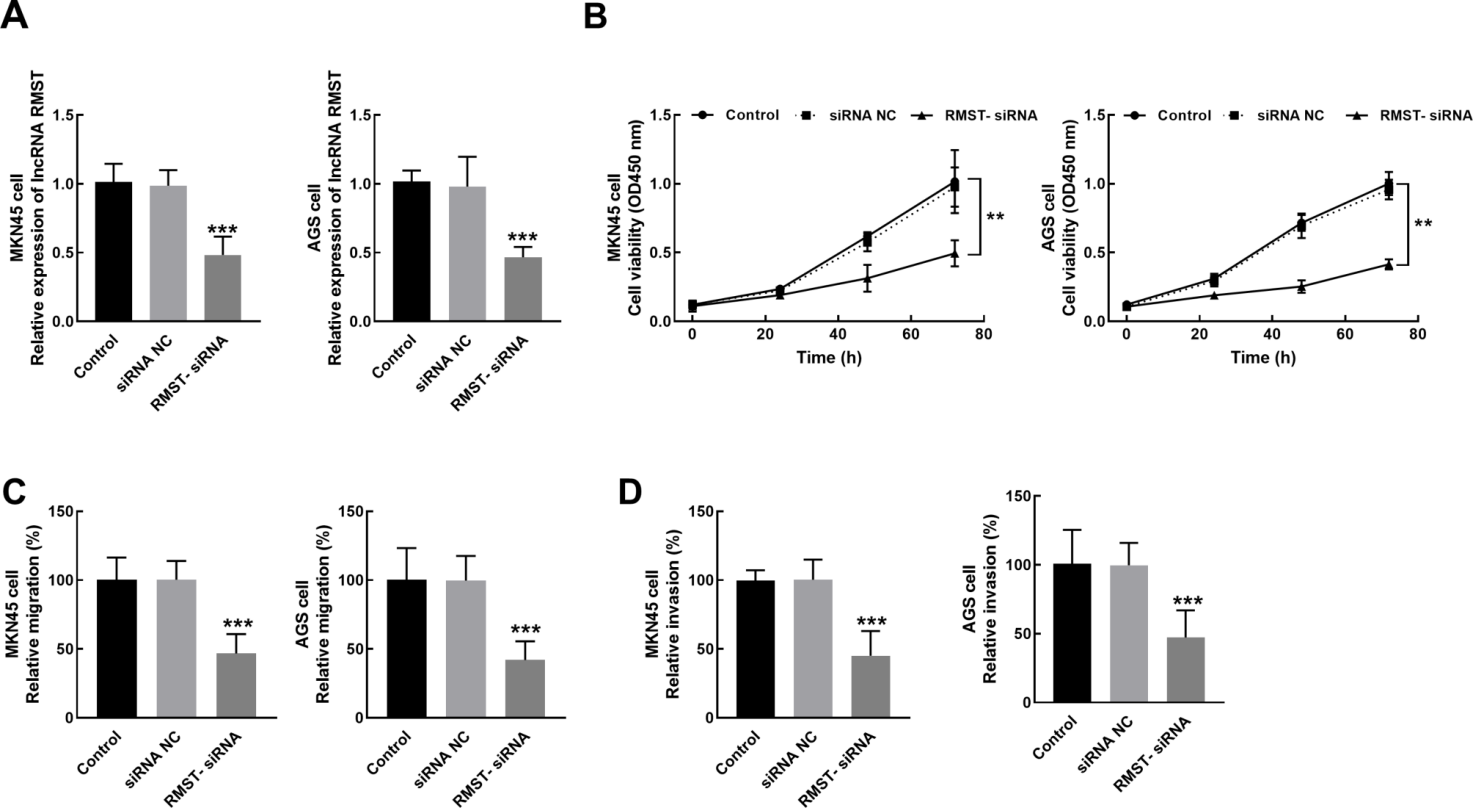
The transfection of RMST small interference RNA significantly suppressed the expression of RMST in MKN45 cells and AGS cells (**A**). The knockdown of RMST significantly suppressed the proliferation (**B**), migration (**C**), and invasion (**D**) of MKN45 and AGS cells. **P < 0.01, ***P < 0.001

RMST was significantly suppressed by the transfection of its small interference RNA in MKN45 and AGS cells (*P* < 0.001, Fig. 3A). The knockdown of RMST markedly inhibited proliferation (*P* < 0.01, Fig. 3B), migration (*P* < 0.001, Fig. 3C), and invasion (*P* < 0.001, Fig. 3D) of MKN45 and AGS cells.

**Fig. 4:**
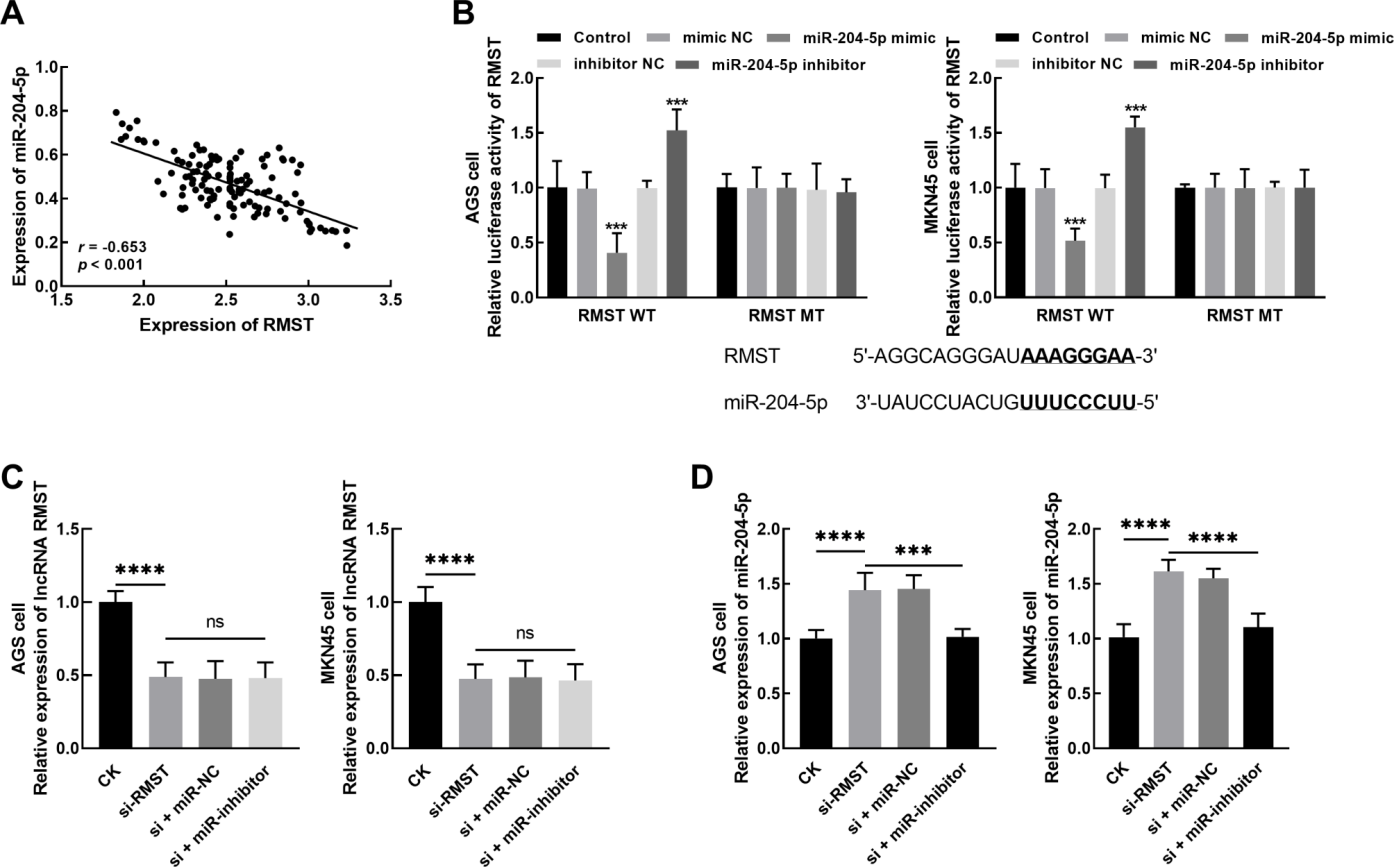
The expression of miR-204-5p in gastric cancer tissues was negatively correlated with the expression of RMST (**A**). The luciferase activity of RMST was significantly suppressed by the overexpression of miR-204-5p and enhanced by its downregulation (**B**). miR-204-5p showed no significant effect on RMST (**C**) but could reverse the enhanced effect of RMST knockdown (D). ^ns^*P* > 0.05, ***P < 0.001, ****P < 0.0001

**Fig. 5:**
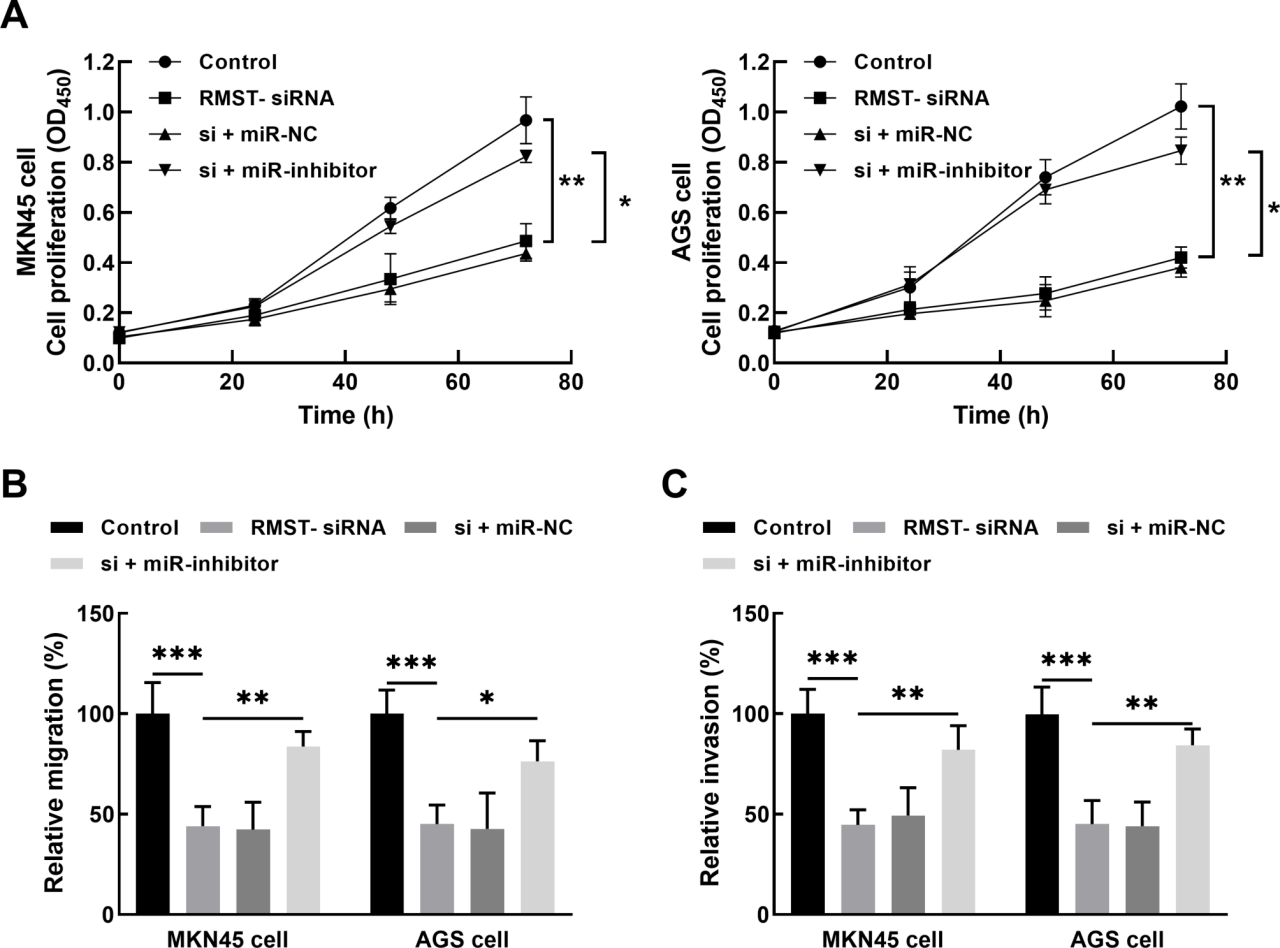
Silencing miR-204-5p could alleviate the inhibitory effect of RMST knockdown on the proliferation (**A**), migration (**B**), and invasion (**C**) of AGS and MKN45 cells. *P < 0.05, **P < 0.01, ***P < 0.001

The expression of RMST showed a negative correlation with miR-204-5p expression in collected tumor tissues (*r* = -0.653, *P* < 0.001, Fig. 4A). In vitro, the lncRNASNP v3 online database (http://gong_lab.hzau.edu.cn/lncRNASNP3#!/) was employed to predict the binding sites between RMST and miR-204-5p. Overexpression of miR-204-5p significantly suppressed the luciferase activity of RMST. The mutant type of RMST was unaffected by the expression of miR-204-5p (*P* > 0.05, Fig. 4B). Additionally, regulation of miR-204-5p showed no significant effect on RMST after its knockdown (*P* > 0.05, Fig. 4C). Silencing RMST dramatically enhanced the expression of miR-204-5p in AGS and MKN45 cells, which was reversed by miR-204-5p inhibitor (*P* < 0.001, Fig. 4D). Moreover, miR-204-5p knockdown alleviated the inhibitory effects of RMST silencing on the proliferation (*P* < 0.05, Fig. 5A), migration (*P* < 0.05, Fig. 5B), and invasion (*P* < 0.05, Fig. 5C) of AGS and MKN45 cells.

## Discussion

Recently, lncRNAs garnered significant interests and emerged as a focal point of research, particularly in the context of human diseases [13]. Several lncRNAs were identified as biomarkers for indicating the development of malignant tumors and predicting patients’ prognosis. For example, lncRNA PTCSC3 could distinguish gastric cancer patients from healthy individuals and predict the poor overall survival of patients [14]. Similarly, lncRNA XLOC_009167 could differentiate lung cancer patients from benign lung disease and healthy persons [15]. Additionally, lncRNA FAM83A-AS1exhibited upregulation in esophageal cancer and stimulated malignant progression [16]. RMST, known for its inhibitory effects on the mitophagy of glioma cells, holds promise as a prognostic factor for patients with glioma [17]. In this study, the upregulation of RMST was closely correlated with advanced TNM stage and lymph node metastasis in patients with gastric cancer, indicating its involvement in cancer progression. Moreover, RMST serves as an indicator of patients’ survival, as its upregulation is consistently correlated with the poor prognosis of gastric cancer. However, the efficacy of postoperative therapy might affect the prognosis of patients. Due to the diverse disease conditions among study subjects, ensuring uniformity in postoperative treatments is of great challenges, which may potentially affect the prognostic value of RMST. Therefore, further investigations should explore the role of RMST in predicting the prognosis of patients receiving various postoperative treatments.

Previous studies have shown the involvement of RMST in various pathological processes of human diseases. Its overexpression accelerates neuronal apoptosis and promotes the M1 polarization of microglial cells [18]. Conversely, the knockdown of RMST impeded neuronal apoptosis and mitigated oxidative stress induced by oxygen and glucose deprivation/reperfusion [19]. Here, RMST was observed to regulate cellular processes in gastric cancer, with its knockdown significantly suppressing such processes. This underscores the tumor promoter role of RMST in gastric cancer.

The modulation of microRNAs represents a widely accepted mechanism through which lncRNAs exert their function [20]. In previous studies, certain miRNAs have been proposed as the targets of RMST, implicating RMST in disease development. For instance, RMST was reported to regulate miR-5692, and its protective effect against myocardial infarction was correlated with the knockdown of miR-5692 [21]. RMST promoted oxygen-glucose deprivation-induced neuronal apoptosis by regulating the hnRNPK/p53/miR-107/Bcl2l2 axis [22]. The interaction between RMST and miR-204-5p was observed in an oxygen-glucose deprivation-induced ischemic stroke model, which attenuated the injury of brain microvascular endothelial cells [11]. Early studies have revealed that miR-204-5p was downregulated in gastric cancer and suppressed cell migration and invasion [12]. miR-204-5p has been proposed as a potential biomarker for distinguishing and monitoring the development of gastric cancer [23].

miR-204-5p was regulated by various lncRNAs, thereby influencing tumor progression in human cancers. For example, lncRNA LINC01234 acted as a tumor promoter in gastric cancer by regulating miR-204-5p [24]. The promoting effect of lncRNA NEAT1 on docetaxel resistance in prostate cancer was correlated with the inhibition of miR-204-5p [25]. The interaction between miR-204-5p and RMST was also validated in the present study. In gastric cancer tissues, miR-204-5p was negatively correlated with RMST expression. Overexpression of RMST significantly reduced miR-204-5p levels in gastric cancer. Additionally, silencing miR-204-5p alleviated the inhibitory effects of RMST on gastric cancer cells, suggesting that modulating miR-204-5p may be the underlying mechanism driving the tumor promoter role of RMST in gastric cancer.

## Conclusions

Taken together, RMST serves as a biomarker for gastric cancer, screening disease development, and predicting the prognosis of patients. Moreover, RMST plays a role of tumor promoter, driving the progression of gastric cancer by modulating miR-204-5p.

## Methods

### Study participants

One hundred and twenty-one patients with gastric cancer who received surgical resection at Affiliated Fuzhou First Hospital of Fujian Medical University were enrolled from January 2013 to December 2015. All patients provided informed consent, and approval was obtained from the Ethics Committee of Affiliated Fuzhou First Hospital of Fujian Medical University. Paired normal and tumor tissues were collected during surgery and verified by pathologists. These collected tissues were stored at -80 °C for subsequent analyses. Survival data for the enrolled patients were obtained by a 5-year follow-up survey conducted via telephone or outpatient reviews.

### Real time-qPCR

Total RNA was extracted using the TRIzol reagent (Invitrogen, USA), and the purity was evaluated with a spectrophotometer (IMPLEN, USA). The concentration of isolated RNA ranged 300–500 mg/µL, with the purity valuated by the ratio of OD260/280 ranging from 1.8 to 2.2 (to eliminate protein contamination) and the ratio of OD260/230 over 1.8 (to exclude residual organic reagent). RNA integrity number (RIN) of RNA over 7.0 was deemed suitable for PCR. Subsequently, cDNA was generated from the isolated RNA using the PrimeScript RT kit (TaKaRa, Japan) for RMST and the TaqMan MicroRNA Reverse Transcription kit (Applied Biosystems, USA) for miR-204-5p. The expression of RMST and miR-204-5p was assessed with the Applied Biosystems 7500 Fast Real-Time PCR System (Applied Biosystems, USA) and the SYBR Green Master (Roche, Germany). The relative expression was calculated with the 2^−△△Ct^ method and normalized to GAPDH (for RMST) or U6 (for miR-204-5p) [26–28]. The sequences of experimental primers are summarized in Table S1.

### Cell culture and cell transfection

Gastric cancer cells (AGS, MKN-45, HGC-27, and NUGC-3 cells) and a normal gastric mucosa cell (GES1) were obtained from ATCC. These cells were cultured at 37 °C with 5% CO_2_ in the RPMI1640 culture medium (containing 10% FBS, Gibco, USA). The cells were available for the following experiments until growing to the logarithmic period.

Then, cells were transfected with RMST small interference RNA, miR-204-5p mimic, miR-204-5p inhibitor, or negative controls to regulate the expression of RMST and miR-204-5p in gastric cancer cells. Cell transfection was conducted with the Lipofectamine 2000 (Invitrogen, USA), and the transfection efficiency was evaluated by RMST and miR-204-5p expression.

### Cell proliferation assay

Cells were seeded into the 96-well plates and incubated at 37 °C with 5% CO_2_ for 0, 24, 48, and 72 h. Subsequently, the CCK8 kit was added to each well and incubated for an additional hour. The absorbance at 450 nm was detected by a microplate reader with a background control serving as the blank.

### Cell migration and invasion assay

Cells (1 × 10^4^ cells/well) were seeded into the upper chamber of the transwell plates and supplied with the FBS-free culture medium. Matrigel was applied to in the upper chamber before the invasion assay. The complete culture medium (supplemented with 10% FBS) was used as a chemoattractant and placed in the bottom chamber. The transwell plates were then incubated at 37 °C for 24 h, and the migrated and invaded cells were counted with a microscope.

### Luciferase reporter assay

The wild-type RMST plasmids were constructed by inserting the fragment containing the binding sites between miR-204-5p and RMST, while the mutant type RMST plasmids were established by the point mutation in the binding sites. The established plasmids were co-transfected with miR-204-5p mimic, miR-204-5p inhibitor, or corresponding negative controls. The luciferase activity was detected with the Synergy 2 Multidetection Microplate Reader (Bio Tek Instruments) and normalized with Renilla.

### Data analysis

All experiments and analyses were performed in triplicate. Results are presented as mean ± SD. Statistical differences were evaluated by Student’s t-test or one-way ANOVA followed by the Turkey post-hoc test. The significance of RMST in disease development was assessed by analyzing its association with patients’ clinicopathological features with the Chi-square test. Furthermore, the clinical significance of RMST was assessed with the Chi-square test, Kaplan-Meier analysis followed by the log-rank test, and the Cox regression analysis. The correlation between tissue RMST level and tissue miR-204-5p was assess by Pearson correlation analysis. Statistical significance was defined as *P* < 0.05.

### Electronic supplementary material

Below is the link to the electronic supplementary material.

Supplementary Material 1

## Data Availability

The datasets used and/or analysed during the current study are available from the corresponding author on reasonable request.

## Abbreviations

lncRNAs: long non coding RNAs
miRNAs: microRNAs
RIN: RNA integrity number
RMST: rhabdomyosarcoma 2-associated transcript
