# Supplementary material for: lncRNA RMST is associated with the progression and prognosis of gastric cancer via miR-204-5p
Source: Cell Div. 2024 Apr 12;19:12. doi: 10.1186/s13008-024-00117-x (PMC11015603; doi:10.1186/s13008-024-00117-x)
Supplement: Supplementary file 1 — Supplementary Material 1 [file 13008_2024_117_MOESM1_ESM.docx]

| Primer | Sequences (5’-3’) |
| --- | --- |
| RMST forward | AGCAATGCATTCTTTCACATGG |
| RMST reverse | ATGCAATTTCGGTGGTTGGC |
| miR-204-5p forward | ACACTCCAGCTGGGTTCCCTTTGTCATCCTAT |
| miR-204-5p reverse | CTCAACTGGTGTCGTGGA |
| GAPDH forward | CCCTCAATGACCACTTTGTGAA |
| GAPDH reverse | AGGCCATGTGGACCATGAG |
| U6 forward | GCTTCGGCAGCACATATACT |
| U6 reverse | GTGCAGGGTCCGAGGTATTC |

Table S1. Primer sequences used in PCR assay
